# Draft Genome Sequence of the Cyanobacterium *Synechococcus* sp. Strain Nb3U1

**DOI:** 10.1128/mra.00251-22

**Published:** 2022-04-19

**Authors:** Christopher L. Pierpont, Satoshi Ohkubo, Hideaki Miyashita, Scott R. Miller

**Affiliations:** a Division of Biological Sciences, University of Montana, Missoula, Montana, USA; b Graduate School of Life Sciences, Tohoku University, Sendai, Aoba-ku, Japan; c Graduate School of Human and Environmental Studies, Kyoto University, Kyoto, Sakyo-ku, Japan; University of Delaware

## Abstract

We report the 3.5-Mb draft genome sequence of the cyanobacterium *Synechococcus* sp. strain Nb3U1, which was isolated from a microbial mat sample collected from Nakabusa Hot Spring, Nagano, Japan.

## ANNOUNCEMENT

*Synechococcus* sp. strain Nb3U1 was isolated from a microbial mat sample collected from Nakabusa Hot Spring, Nagano, Japan; mat homogenates were grown in BG11 medium under far-red light, and individual cyanobacterial cells were isolated microscopically from these enrichments (1). 16S rRNA sequence analysis identified Nb3U1 as a member of lineage T1 (2). T1 cyanobacteria are a cosmopolitan but poorly understood group from mesothermic to moderately high-temperature habitats in alkaline geothermal environments. Notably, T1 cyanobacteria appear to be the less-thermotolerant sister taxa of the *Synechococcus* A/B clade, members of which define the upper temperature limit for phototrophy on Earth (~74°C [3]).

Strain Nb3U1 was grown in liquid BG11 medium under ~75 μmol photons m^−2^ s^−1^ of cool white fluorescent light with a 12-h/12-h photoperiod. Genomic DNA (gDNA) for paired-end, short-read sequencing was extracted from the growing cells using Qiagen’s DNeasy PowerBiofilm kit. A library was prepared using a Nextera DNA Flex kit, followed by 150 cycles of paired-end sequencing on an Illumina NextSeq 550 platform. High-molecular-weight gDNA for long-read sequencing was later extracted from the same culture using Qiagen’s Genomic-tip 20/G protocol, prepared using the Nanopore ligation sequencing kit without shearing or size selection and sequenced for 48 h using a Nanopore MinION sequencer (FLO-MIN106D, R9.4.1); GUPPY v4.5.4 (https://staff.aist.go.jp/yutaka.ueno/guppy/) was used for Nanopore base calling. Prior to sequencing, DNA quality and quantity were assessed by spectrophotometry (Agilent Tape Station) and fluorimetry (Qubit 2.0), respectively. The quality of all sequenced reads was checked using FastQC v0.11 (https://www.bioinformatics.babraham.ac.uk/projects/fastqc/), but none were removed. A hybrid draft assembly using 8,011,914 paired-end Illumina short reads (35 to 151 bp) and 141,038 Nanopore long reads (83 to 51,308 bp) was built *de novo* using SPAdes v3.12.0 (4). Following the assembly, contigs shorter than 1 kbp were removed prior to subsequent analyses. Kraken v2.1.2 (5) and BLAST+ v2.2.31 (6) were used to identify and extract contigs with high sequence similarity to cyanobacteria if (i) the contig returned significant local BLAST hits (E values, ≤1e^−100^) to two *Synechococcus* A/B reference genomes (7) and/or (ii) Kraken assigned an NCBI TaxID from the phylum *Cyanobacteria*, the order *Synechococcales*, or either of the above reference genomes.

Genomic statistics for these extracted contigs were measured using QUAST v4.5 (8). The total size of the refined assembly is 3,487,976 bp in 4 contigs, with an *N*_50_ value of 2,215,988 bp, a GC content of 55.19%, and a mean coverage of 73.0×. The Nb3U1 genome was estimated to be 93.8% complete using BUSCO v5.2.2 (9) with reference to the *Synechococcales* lineage. In identical analyses, the closed *Synechococcus* A/B reference genomes were estimated to be 94.4% and 94.9% complete. We therefore conclude that the Nb3U1 genome assembly is essentially complete. The refined assembly was annotated using NCBI’s PGAP software (10). Of the 3,300 predicted features, 3,209 are protein coding, 42 are tRNAs, 4 are noncoding RNAs (ncRNAs), and 3 are rRNAs. All software tools were run with default parameters unless otherwise specified.

### Data availability.

This whole-genome shotgun sequencing project has been deposited at DDBJ/ENA/GenBank under accession number JAKFYQ000000000.1. The version described in this paper is version JAKFYQ010000000. These data are associated with BioProject accession number PRJNA795194 under BioSample accession number SAMN24695622. The raw data from the Illumina and Nanopore sequencing are available under SRA accession numbers SRR17483484 and SRR17483483, respectively.
